# Heart Rate Variability as a Potential Predictor of Response to Intranasal Esketamine in Patients with Treatment-Resistant Depression: A Preliminary Report

**DOI:** 10.3390/jcm13164767

**Published:** 2024-08-14

**Authors:** Lorenzo Moccia, Giovanni Bartolucci, Maria Pepe, Ilaria Marcelli, Flavia Grisoni, Andrea Brugnami, Romina Caso, Francesca Bardi, Claudia Calderoni, Alessandro Michele Giannico, Elisabetta Benini, Marco Di Nicola, Gabriele Sani

**Affiliations:** 1 Department of Psychiatry, Fondazione Policlinico Universitario “A. Gemelli” IRCCS, 00168 Rome, Italy; 2 Department of Neuroscience, Section of Psychiatry, Università Cattolica del Sacro Cuore, 00168 Rome, Italy

**Keywords:** esketamine, biomarkers, mood disorders, personalized medicine

## Abstract

**Background:** Esketamine has received approval as a nasal spray (ESK-NS) for treatment-resistant depression (TRD) and evidence from real-world investigations has confirmed the effectiveness of ESK-NS, albeit with interindividual differences in response. Heart rate variability (HRV), defined as the fluctuation in time interval between consecutive heartbeats, can be used to measure autonomic dysfunction in psychiatric disorders and its role has been investigated in diagnosis and prognosis of depression. **Methods:** This preliminary report aims to evaluate HRV parameters and their association with treatment outcome in 18 patients (55.6% males, 55.6 ± 9.39 years old) with TRD treated with a target dose of ESK-NS for one month (mean dose: 80.9 ± 9.05 mg). The Beck Depression Inventory (BDI) and a 3 min resting electrocardiogram were used to assess changes in depressive symptoms and HRV measurements before and after treatment. **Results:** Responders (n = 8, 44.5%; based on ≥30% BDI scores reduction) displayed lower HRV values than non-responders at baseline (*p* = 0.019), which increased at one month (*p* = 0.038). Receiver–Operating Characteristic (ROC) curves obtained from a logistic regression displayed a discriminative potential for baseline HRV in our sample (AUC = 0.844). **Conclusions:** These preliminary observations suggest a mutual interaction between esketamine and HRV, especially in relation to treatment response. Further studies are required to investigate electrophysiological profiles among predictors of response to ESK-NS and allow for personalized intervention strategies in TRD that still represent a public health concern.

## 1. Introduction

Despite the lack of a universally shared definition, treatment-resistant depression (TRD) represents a relevant public health concern based on its prevalence and socioeconomic impact [1]. Almost one-third of individuals diagnosed with depression experience the persistence of symptoms, despite adequate medication, and available therapeutic options for treatment-resistant depressive episodes are still limited [1,2].

The monoaminergic theory of mood disorders has been progressively expanded with the investigation of other causal hypotheses including the glutamatergic system, especially in relation to TRD [3,4,5]. Esketamine (ESK), the S-enantiomer of ketamine with a strong noncompetitive antagonism of glutamatergic N-methyl-D-aspartate (NMDA) receptors [6], has demonstrated efficacy as a nasal spray (NS) in treating TRD when combined with oral antidepressants in both randomized trials and real-world investigations [7,8,9]. However, the effectiveness of ESK-NS may vary among patients with TRD [10,11,12] and, despite some efforts to identify predictive clinical features [2], potential predictors of treatment response need to be further investigated.

Depression is associated with impaired autonomic function, as evidenced by alterations in heart rate variability (HRV) [13,14,15]. HRV quantifies the extent of variation in the inter-beat (R-R) interval duration derived from an electrocardiogram (ECG) and represents the dynamic interaction between the sympathetic and parasympathetic branches of the autonomic nervous system (ANS). Several studies have reported the presence of lower resting levels of HRV in individuals with depression compared to healthy subjects [13,16,17]. However, the exact mechanisms linking HRV to depression are currently under investigation. There is some evidence showing that a dysregulation in hypothalamic–pituitary–adrenal (HPA) axis activity, as observed in major depression, can disrupt ANS functioning which, in turn, may lead to reduced HRV. Additionally, alterations in central nervous system pathways involved in both depression and autonomic regulation, such as the amygdala and the prefrontal cortex, may contribute to the HRV deficits observed in depressed patients [18]. Furthermore, HRV has been correlated with the intensity of the disease and with fluctuations in the severity of depressive symptoms [19,20].

The effect of antidepressant medications on HRV values has been investigated in multiple studies, albeit with mixed results [21,22]. It has been reported that alterations in baseline measures of HRV in response to emotional triggers are linked to a reduction in depressive symptomatology following fluoxetine intake [23]. Similarly, after two weeks of antidepressant treatment, Hartmann et al. [24] observed a significant enhancement in HRV parameters corresponding to an improvement in depressive symptoms. However, there is also evidence stating that HRV scores may not change following pharmacological interventions, suggesting HRV as a trait—rather than a state—marker for major depression [25].

To our knowledge, the relationship between HRV and ESK-NS administration has not yet been evaluated in TRD. Therefore, this preliminary report aimed to investigate HRV measures and their association with the effects of one-month treatment with esketamine and, additionally, to assess whether these may serve as potential predictors of response.

## 2. Materials and Methods

### 2.1. Participants

Patients accessing the Psychiatric Unit of the Fondazione Policlinico Universitario Agostino Gemelli IRCCS, aged between 18 and 70 years, with a primary diagnosis of major depressive disorder (MDD) based on the criteria of DSM-5-TR [26], and presenting with a treatment-resistant major depressive episode (MDE) according to the European Medicines Agency definition, were screened for inclusion. Patients’ eligibility was established as follows: inadequate response to two or more antidepressant therapies from at least two distinct classes, assumed at a proper dosage for 6 to 8 weeks, during the present episode [27]; at least moderate depression based on Beck Depression Inventory (BDI) total score > 19; current antidepressant treatment with selective serotonin reuptake inhibitors (SSRIs) and/or serotonin/norepinephrine reuptake inhibitors (SNRIs) at stable dosages during the previous six weeks; fluency in spoken and written Italian.

Current medical or psychiatric contraindications to administration of esketamine (like pregnancy, cardiovascular disorders, substances/alcohol use, psychotic symptoms, neurological diseases), impairment of cognitive functioning detected by a Mini-Mental State Examination (MMSE) score < 26 [28], and current intake of medications that could affect cardiorespiratory activity [29,30] were the exclusion criteria. Moreover, given evidence suggesting that regular exercise can influence cardiac vagal tone [31,32], only patients who were not regularly engaged in athletic or endurance sports were enrolled. According to the previous criteria, a final sample of 18 patients (10 males, 8 females) was recruited (a summary of sociodemographic and clinical characteristics is reported in Results, Table 1).

### 2.2. Procedures

The treatment protocol consisted of the administration of ESK-NS in addition to ongoing oral antidepressants, twice a week for a month and with the initial dosage of either 28 or 56 mg depending on the cut-off age of ≥65 or <65 years, respectively. Subsequent intakes were gradually increased up to 56 or 84 mg according to tolerability and clinical judgement, with 84 mg as the maximum dosage independent of age. No adjustments based on body weight or renal functioning are required during treatment with ESK-NS, though caution in administering 84 mg is suggested in cases of moderate hepatic impairment [EMA, http://www.ema.europa.eu/, accessed on 1 August 2024]. Here, all patients reached 84 mg at one month (mean final dose: 80.9 ± 9.05 mg) except two because of tolerability issues and medical comorbidities. Concomitant medications, such as augmentation with mood stabilizers and/or antipsychotics, were allowed and were not modified all across the observation period.

Sociodemographic (i.e., age, education, employment, gender, marital status) and clinical information (such as body mass index, number of lifetime MDEs, smoking) was collected. Current antidepressant treatments have been reported as fluoxetine equivalent doses.

Trained raters conducted diagnostic interviews upon entry in the study demonstrating high inter-rater reliability (k > 0.8). The Structured Clinical Interviews for DSM-5 Clinician Version (SCID-5-CV) and Personality Disorders (SCID-5-PD) [33,34] were used to confirm psychiatric diagnoses. The self-administered BDI was completed by patients at baseline and after one month of treatment. Depressive symptoms severity was investigated through the total scores of BDI [35] and patients were considered responders if obtaining a ≥30% reduction in baseline BDI scores at one month [36].

#### HRV and ECG Recording

To assess changes in HRV, patients underwent a 3 min resting electrocardiogram (ECG) before starting ESK-NS administration and after four weeks of treatment. Each participant was equipped with three 10 mm Ag/AgCl pre-gelled electrodes (ADInstruments, Oxford, UK), positioned on the wrists in an Einthoven’s triangle configuration for ECG recording. Then, ECG data were converted and amplified using an eight-channel amplifier (PowerLabT26; ADInstruments, UK) and subsequently displayed, stored, and analyzed through the LabChart 7.3.1 software package (ADInstruments Inc., 2011). Participants were seated in a quiet, well-lit environment and were advised to relax and stay still throughout the recording to reduce the occurrence of motion artifacts.

ECG data were sampled at a frequency of 1 kHz and filtered online using the Mains Filter. The peak of the R-wave in the ECG was identified for each consecutive heartbeat, and the R-R interval was measured to the nearest millisecond. R-R intervals underwent examination and correction for artifacts. The correction process involved the use of software-based artifact detection with the artifact threshold set at 300 milliseconds (LabChart’s ECG Analysis module, ADInstruments Inc., 2011), followed by a visual inspection of the recorded ECG signal. Any identified artifacts were subsequently corrected through integer division or summation [37].

The magnitude of HRV was computed using CMetX (available at http://apsychoserver.psych.arizona.edu, accessed on 29 July 2024). CMetX enables the extraction of cardiac chronotropy metrics, with an inter-beat interval (IBI) series given as input [37]. HRV was operationalized as the natural log of band-limited (0.12–0.40 Hz) variance of IBI time series. This analytic approach aligns with the Porges–Bohrer method for HRV calculation (i.e., *V*-hat). The *V*-hat metric is a hybrid of frequency-method filtering and a time-domain method designed to remove sources of variance in the heart period time series other than the variance within the frequency band of spontaneous breathing [38,39]. This method has been shown to be particularly sensitive to vagal influence on cardiac activity. In addition, the logarithmic transformation adjusts skewness and kurtosis in HRV, making the data more closely conform with the assumption of normality [40,41]. In addition, HRV, as computed with the method of Porges and Boher, demonstrated high intercorrelation with other HRV time-domain metrics that are supposed to reflect vagal influence on cardiac chronotropy, such as RMSSD and pNN50 [42].

HRV values were derived through the following steps: (a) linear interpolation at a sampling rate of 10 Hz; (b) application of a 241-point FIR filter with a bandpass of 0.12–0.40 Hz; (c) extraction of the variance within the bandpass; (d) transformation of the variance into its natural logarithm [43,44]. As per guidelines, this procedure was applied to distinct epochs of 30 s and HRV values were computed as the average across six 30 s epochs [37].

### 2.3. Statistical Analysis

The sample was subdivided into two groups—responders vs. non-responders—according to a one-month ≥ 30% reduction in baseline BDI scores. Comparisons for sociodemographic and clinical features were conducted through contingency tables/χ^2^ and Student’s *t*-test for categorical and continuous variables, respectively. Repeated measures ANOVA was employed to assess responder vs. non-responder differences in HRV measures before and after one month of treatment. The model comprised a between-subject factor (responders vs. non-responders), a repeated measures time factor, and a group-by-time interaction with gender and age added as covariates. Following significant effects and interactions, Tukey-corrected post-hoc tests were employed. Effect sizes were reported using partial eta-squared (η^2^p), with values of 0.01, 0.06 and 0.14 indicating, respectively, small, medium and large effects [45]. To test the discriminative power of HRV for ESK-NS outcome, we performed a logistic regression analysis to detect odds ratios (ORs) and 95% confidence intervals (CIs) using baseline HRV measures (i.e., parameters obtained before the first esketamine administration) and responders vs. non-responders as the dependent variable. Receiver–Operating Characteristic (ROC) curves were also obtained for baseline HRV. A level of *p* < 0.05 was used for establishing statistical significance. All analyses were conducted through IBM SPSS Statistics for Windows, v. 28 (IBM Corp., Armonk, NY, USA).

## 3. Results

All sociodemographic and clinical characteristics at baseline are reported in Table 1.

**Table 1:** Sociodemographic and clinical characteristics at baseline.

*Characteristics* (n, %; M ± SD)	Overall	Responders	Non-Responders	t/χ^2^/F	*p*
**Overall**	18	8 (44.5)	10 (55.5)		
*Sociodemographic features*					
**Age** (years)	55.6 ± 9.39	51 ± 9.4	59.2 ± 8.05	1.99	0.06
**Gender**				0.18	0.67
Male	10 (55.6)	4 (50)	6 (60)		
Female	8 (44.4)	4 (50)	4 (40)		
**Education** (years)	14.7 ± 2.97	14.9 ± 2.6	14.5 ± 3.4	−0.26	0.79
**Occupation** (unemployed)	10 (55.6)	4 (50)	6 (60)	0.18	0.67
**Marital status** (unmarried)	8 (44.4)	3 (37.5)	5 (50)	0.28	0.59
*Clinical data*					
**BMI**	26.9 ± 4.43	26.8 ± 5.21	26.9 ± 3.99	0.02	0.99
**Smoking**	8 (44.4)	4 (40)	4 (50)	0.18	0.67
**Lifetime MDE** (number)	3.78 ± 2.26	3.75 ± 2.92	3.8 ± 1.75	0.05	0.96
**FLX equivalents** (mg)	56.5 ± 23.1	60.6 ± 23.8	54.5 ± 24.1	−0.41	0.69
**BDI**	35.7 ± 9.1	37.1 ± 10.5	34.4 ± 8.02	−0.59	0.56
**HRV** (lnmsec)^2^	6.01 ± 1.1	5.37 ± 0.93	6.52 ± 0.91	2.62	**0.02**

Significant results are in **bold**. Abbreviations: BDI, Beck Depression Inventory; BMI, body mass index; F, Fisher’s exact test; FLX equivalents, fluoxetine equivalent; HRV, heart rate variability; M, mean; MDE, major depressive episode; mg, milligram; lnmsec, natural logarithm millisecond; n, number; *p*, statistical significance; SD, standard deviation; t, Student’s t; χ^2^, chi-squared test.

At one month, we observed an overall reduction in BDI total scores (28.7 ± 12.58), with 8 (44.5%) patients rated as responders and 10 (55.5%) as non-responders (BDI: 23.5 ± 11.7 vs. 32.9 ± 12.1; *p* = 0.115). There were no significant between-group differences as for mean ESK-NS dose (80.5 ± 9.9 vs. 81.2 ± 8.85; *p* = 0.88) nor for other antidepressant treatments converted into fluoxetine equivalent dosages [46].

Repeated measures ANOVA revealed a significant effect for group (responders vs. non-responders, *p* = 0.013), age (*p* = 0.014), and time × group interaction (*p* = 0.002), without a significant effect for time (*p* = 0.940) and gender (*p* = 0.123) (Table 2).

After post hoc correction of *t*-tests comparing baseline vs. one-month HRV in responders vs. non-responders, significantly lower HRV values at baseline were detected in responders compared to non-responders [5.37 ± 0.93 ln(msec)^2^ vs. 6.52 ± 0.91 ln(msec)^2^; t = 3.69, *p* = 0.011] and no differences were detected at one-month follow-up [6.04 ± 0.72 ln(msec)^2^ vs. 6.04 ± 0.80 ln(msec)^2^; t = 0.941, *p* = 0.784] (all post hoc comparisons are shown in Table 3).

**Figure 1:**
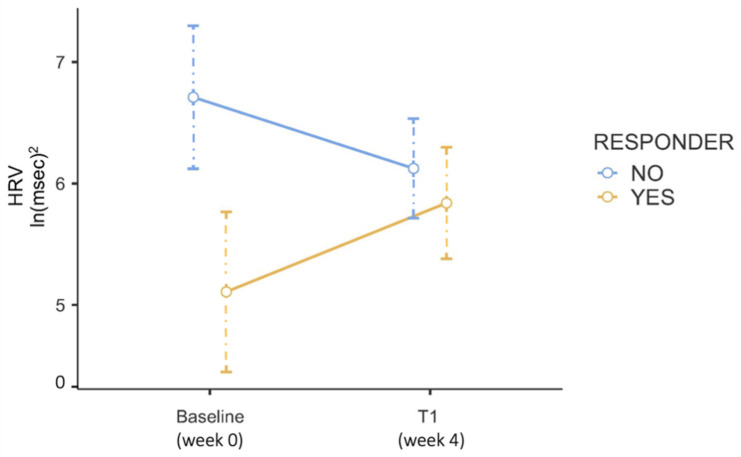
Repeated measures ANOVA showing heart rate variability (HRV) changes between baseline and one month in responders (yellow line) vs. non-responders (blue line).

A significant HRV increase from baseline to one month was observed in responders (t = −3.06, *p* = 0.038), but not in non-responders (t = 2.74, *p* = 0.067) (Figure 1).

**Figure 2:**
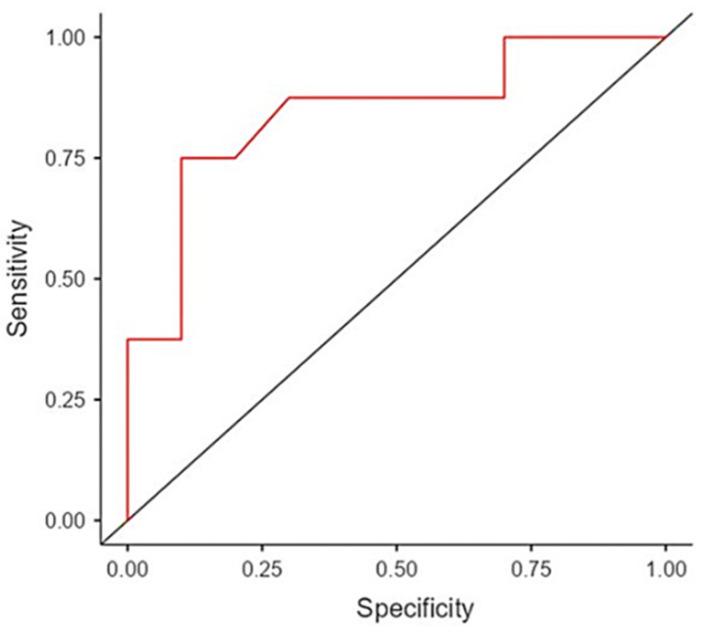
The Receiver–Operating Characteristic (ROC, red) curves display the predictive capacity of the activity of the autonomous nervous system (ANS) in esketamine treatment for MDD, assessed before infusion (i.e., baseline) through HRV parameters.

The logistic regression showed that baseline HRV was significantly associated with treatment response (SE = 0.701, OR [95% LLCI-ULCI] = 0.244 (0.06–0.96), *p* = 0.04). The area under the curve (AUC) was 0.844, and showed a sensitivity of 75%, specificity of 92% and accuracy of 83.3%. These results showed that baseline HRV, as a predictive marker of response, had some discriminative potential in our sample (Figure 2).

## 4. Discussion

This preliminary report shows that responders to esketamine—established according to a 30% reduction in BDI scores after one month of treatment—exhibited lower HRV compared to non-responders at baseline but not at follow-up. We also found that HRV significantly increased between baseline and one-month timepoint in the responder-group, supporting the hypothesis that HRV can be modified following esketamine administration in patients who respond to antidepressant treatment, similarly to other pharmacological compounds. Moreover, HRV obtained before ESK-NS administration significantly predicted treatment response one month later.

Research has progressively highlighted the complex interplay between mental illnesses and ANS functioning, including diminished vagal (parasympathetic) modulation and heightened sympathetic activity [47]. Specifically, studies have consistently reported an association between depression and such dysregulation that usually reflects in increased susceptibility towards cardiac morbidity and mortality [48]. From this perspective, HRV shows potential in clarifying the relationship between MDD and autonomic dysregulation, as well as in enhancing our comprehension of the connection between emotional reactivity and physiological adaptability [47,48]. The Neurovisceral Integration Model, introduced by Thayer and Lane [49], is a prominent bio-behavioral framework that posits vagally mediated HRV as an indicator of vagal inhibition of the heart. This serves as the primary output of the central autonomic network, which is responsible for managing psychophysiological resources and responding to environmental demands [50]. Within this model, higher HRV reflects a healthy, adaptive organism with improved self-regulation abilities [49]. Elevated resting-state HRV is associated with positive psychological outcomes, whereas lower resting-state HRV correlates with symptoms of internalizing and externalizing psychopathology [50]. Thus, HRV may be considered a psychophysiological marker for psychopathology.

Research has shown that HRV is linked to self-regulatory processes, encompassing physiological and emotional regulation as well as cognitive functioning, with various brain regions identified as potential neural correlates [51]. Indeed, an integrated network involving both cortical (like cingulate, insula and prefrontal) and subcortical (like hippocampus, midbrain and striatum) brain areas, as well as the amygdala, seems to modulate HRV, which can serve as an index of a complex neurobiological pathway that controls emotional, cognitive, physiological, and behavioral responses to emotional stimuli [51,52].

It is noteworthy that MDD is characterized by a reduced global brain connectivity with a mix of cortical hypoactivity and subcortical hyperactivity [24,53]. A dysfunctional coordination from subcortical structures of the three different autonomic systems, i.e., the sympathetic, the parasympathetic associated with vegetative responses and the parasympathetic linked to social engagement, may result in distinct phenotypes of depression [24]. Given evidence reporting the persistence of sustained low HRV in depressed patients regardless of antidepressant medication and clinical outcome, it could be argued that similar reductions may be attributed to intrinsic MDD pathophysiological mechanisms and that HRV might be proposed as a trait-marker of depression preferentially linked with a distinct depressive phenotype [25].

Administration of ketamine was shown to differentially modify brain connectivity depending on regions, either improving or reducing interconnections between cortical (such as prefrontal and cingulate cortices) and subcortical areas (including amygdala) with subsequent distinct effects on treatment outcomes [53]. Functional neuroimaging studies have investigated immediate and delayed changes induced by esketamine infusion in both depressed patients and healthy volunteers and highlighted that brain areas pertaining to the default mode network, to the anterior cingulate and to prefrontal cortices are involved [54].

Within this background, it could be hypothesized that ESK-NS might affect HRV in light of the partial overlap between the brain areas on which esketamine acts and those identified as neural correlates of HRV. In this view, there is some evidence from the literature indicating that antidepressant compounds might improve HRV, usually reduced in patients with MDD. For instance, a relevant normalization trend in HRV parameters has been reported alongside a reduction in depressive symptoms within just two weeks from the beginning of antidepressant treatment [24]. Similarly, pharmacological interventions leading to symptomatic improvement were linked with heightened HRV in a cohort of 17 patients with MDD, suggesting a potential connection between antidepressant effectiveness and HRV enhancement [55]. In addition, it has been observed that individuals with a lifetime history of MDE exhibited lower HRV levels [56]. Nonetheless, administration of sertraline led to a significative elevation in HRV, which was correlated with mood enhancement [56], suggesting that the link between HRV, antidepressants, and mood regulation might be quite complex.

Despite the abovementioned, studies examining the predictive role of HRV in MDD are still limited. Fraguas and colleagues [23] explored changes in HRV during an emotional induction task as a potential predictor of response to antidepressant treatment. Their findings emphasized the significance of HRV in predicting response in MDD patients but also highlighted its dynamic nature, observing gradual improvements together with a reduction in depressive symptoms during treatment with SSRIs. Similarly, a randomized, multicenter trial involving 722 patients with MDD highlighted that baseline HRV predicted the antidepressant response when differentiating between anxious and non-anxious depression [57]. In cases of depression with anxiety symptoms, patients with greater HRV experienced more favorable outcomes than those with lower HRV. Conversely, individuals with lower HRV demonstrated better treatment outcomes than those with higher HRV in non-anxious depression [57].

Some limitations should be acknowledged for this report, which suggest caution in drawing conclusions, such as the small sample size, the response criterion of a 30% decrease in endpoint BDI scores (vs. treatment response generally defined for at least a 50% reduction from baseline) and the low generalizability of these findings to patients with TRD from other settings. However, it has to be noted that the 50% threshold may often exclude several patients who actually experience benefits from treatment despite not reaching the required cut-off. In our analysis, lowering this threshold allowed us to include patients who improved within one month of treatment without meeting the traditional criteria for response. Furthermore, patients from this study were concurrently taking antidepressant medications, which makes it challenging to distinguish the effect of ESK-NS from that of other compounds on ANS activity, including HRV. Nonetheless, this setting closely mirrors real-world conditions where patients with TRD generally consume multiple medications simultaneously. In light of this, including a control group with healthy subjects could assist the evaluation of the interaction between HRV and esketamine without additional, possible confounding factors, and future studies with a similar design are, indeed, encouraged. Finally, the relatively short-term (i.e., one month) follow-up needs to be mentioned. Despite the rapid antidepressant effect detected for ESK-NS in registration trials (i.e., within the four-weeks induction phase), recent evidence depicted a greater response at three months in real-world samples [7], both sustaining the long-term efficacy of ESK-NS continuation and highlighting the need for longitudinal studies with longer follow-ups to investigate HRV changes and their predictive value. Nevertheless, there are no other prospective studies assessing the impact of treatment with ESK-NS on HRV in a well-selected cohort of patients with TRD, suggesting that pre-treatment HRV may be an outcome predictor. Our findings support and expand previous results reporting that subjects who positively respond to a single intravenous dose of ketamine in MDD display significantly lower baseline HRV compared to non-responders and that HRV significantly increases following ketamine infusion in depressed patients [36].

HRV has been demonstrated to have a good screening sensitivity in several mental health conditions beyond affective disorders [58,59,60], possibly higher than standard screening measures based on subjective self-reports [61,62]. Accordingly, its predictive value of treatment response has also been reported for therapeutic approaches other than antidepressant compounds such as cognitive behavioral therapy [63] and transcranial magnetic stimulation techniques [59]. Therefore, further research is needed to spread insight about the value of low-cost and technically simple predictive biomarkers, like HRV, and to support their integration in routine clinical practice as a tool for better characterizing patients’ phenotypes as well as predicting treatments outcome [64,65]. Moreover, randomized controlled trials are needed to investigate the role of HRV parameters as a therapeutic target per se given available evidence supporting an improvement of psychopathology and stress-related symptoms after HRV biofeedback techniques [66,67].

In conclusion, to our knowledge, this is the first report suggesting that HRV, a cardiac index reflecting ANS functioning, may discriminate between patients with TRD who respond to ESK-NS and those who do not, and, thus, proposing HRV as a useful tool for identifying individuals who are more likely to respond to this type of treatment. These observations might contribute to precision medicine strategies that enhance prediction of treatment outcomes by integrating both clinical and biological evaluations. Investigating HRV could assist in identifying patients with TRD who are more likely to respond, and in a relatively short time, to intranasal esketamine as well as in promptly recognizing those who may benefit from alternative therapeutic interventions.

## Figures and Tables

**Table 2:** Changes in HRV reactivity during treatment by age and gender as covariates (repeated measures ANOVA).

	F	*p*	η^2^p
*Within Subjects Effects*			
**Time**	0.006	0.940	0.00
**Time × Age**	0.663	0.429	0.04
**Time × Gender**	3.421	0.086	0.19
**Time × Group**	15.24	**0.002**	0.52
*Between Subjects Effects*			
**Age**	7.91	**0.014**	0.36
**Gender**	2.7	0.123	0.16
**Group**	7.98	**0.013**	0.36

Significant results in **bold**. Abbreviations: η^2^p, partial eta-squared; F, between- and within-group ratio; *p*, statistical significance.

**Table 3:** Tukey-corrected post hoc comparison of HRV changes after one month of treatment with esketamine nasal spray in responders and non-responders.

HRV	Group	HRV	Group	*Mean Change (SE)*	*p*
*Baseline*	Non-responders	*Baseline*	Responders	1.602 (0.434)	**0.011**
	Non-responders	1 *month*	Non-responders	0.59 (0.21)	0.06
	Non-responders		Responders	0.871 (0.360)	0.119
	Responders		Non-responders	−1.016 (0.373)	0.070
	Responders		Responders	−0.73 (0.239)	**0.038**
*1 month*	Non-responders		Responders	0.285 (0.303)	0.784

Significant results in **bold**. Abbreviations: HRV, heart rate variability; SE, standard error; *p*, statistical significance.

## Data Availability

Authors do not have permission to share these data.
